# Pridopidine, a dopamine stabilizer, improves motor performance and shows neuroprotective effects in Huntington disease R6/2 mouse model

**DOI:** 10.1111/jcmm.12604

**Published:** 2015-06-22

**Authors:** Ferdinando Squitieri, Alba Di Pardo, Mariagrazia Favellato, Enrico Amico, Vittorio Maglione, Luigi Frati

**Affiliations:** IRCCS NeuromedPozzilli, Italy

**Keywords:** pridopidine, Huntington disease, neuroprotection, BDNF, Sig-1R

## Abstract

Huntington disease (HD) is a neurodegenerative disorder for which new treatments are urgently needed. Pridopidine is a new dopaminergic stabilizer, recently developed for the treatment of motor symptoms associated with HD. The therapeutic effect of pridopidine in patients with HD has been determined in two double-blind randomized clinical trials, however, whether pridopidine exerts neuroprotection remains to be addressed. The main goal of this study was to define the potential neuroprotective effect of pridopidine, in HD *in vivo* and *in vitro* models, thus providing evidence that might support a potential disease-modifying action of the drug and possibly clarifying other aspects of pridopidine mode-of-action. Our data corroborated the hypothesis of neuroprotective action of pridopidine in HD experimental models. Administration of pridopidine protected cells from apoptosis, and resulted in highly improved motor performance in R6/2 mice. The anti-apoptotic effect observed in the *in vitro* system highlighted neuroprotective properties of the drug, and advanced the idea of sigma-1-receptor as an additional molecular target implicated in the mechanism of action of pridopidine. Coherent with protective effects, pridopidine-mediated beneficial effects in R6/2 mice were associated with an increased expression of pro-survival and neurostimulatory molecules, such as brain derived neurotrophic factor and DARPP32, and with a reduction in the size of mHtt aggregates in striatal tissues. Taken together, these findings support the theory of pridopidine as molecule with disease-modifying properties in HD and advance the idea of a valuable therapeutic strategy for effectively treating the disease.

## Introduction

Huntington disease (HD) is a neurodegenerative, dominantly transmitted disease whose single *HTT* gene mutation results in the synthesis of mutant huntingtin (mHtt), a misfolded protein, with an expanded polyglutamine stretch in the N-terminus 1. The resulting mutant protein causes a cascade of toxic events in the nervous system, which lead to neuronal cell dysfunction and death. The wide range of HD-related cellular and neurochemical alterations result in a complex and progressively disabling phenotype, in which hyperkinetic (*i.e*. chorea and dystonia) and hypokinetic (*i.e*. parkinsonisms such as bradykinesia and rigidity) clinical manifestations coexist throughout the disease course. Hyperkinesia generally predominate at the beginning of the clinical course, whilst parkinsonisms are more often observed in the advanced stages of HD. The clinical complexity of such conditions seems to be linked to aberrant dopaminergic transmission in HD 2,3 and represents a challenge for effectively treating the disease. In patients with HD, the guiding hypothesis is that pre-synaptic activation of the nigrostriatal dopamine (DA) pathway induces chorea while loss of DA inputs induces bradykinesia 4,5, thus giving rise to the biphasic motor symptoms of early and late HD; hence either abnormally reinforcing or reducing dopaminergic effects may worsen the symptoms severity and disease progression.

Although the dopaminergic circuitry in HD is thought to be complex, current opinions support its relevance as therapeutic target and give rise to the hypothesis that DA stabilizers may provide new valuable treatment options.

Pridopidine, a DA stabilizer, which belongs to a new family of compounds known as dopidines, modulates DA transmission and regulates both hyper- and hypoactive functioning depending on the prevailing dopaminergic tone 6. Pridopidine has been previously tested in patients with Parkinson's disease 7, schizophrenia 8 and is currently in development for the symptomatic treatment of HD. Recent clinical studies identified pridopidine as a molecule with promising therapeutic potential for patients with HD 9–11. In two double-blind randomized phase II (HART study) and phase III (MermaiHD study) clinical trials, pridopidine has been shown to improve overall motor function, as measured by the Unified Huntington's disease Rating Scale (UHDRS) total motor score (TMS) 9,11, and to display a favourable safety and tolerability profile in patients with HD, even after 1 year treatment 10.

Although the favourable profile, the intriguing abilities to modulate DA-related behaviour and the long-term effects that pridopidine has shown in HD patients are encouraging, the exact mechanism by which these effects are induced is not fully understood and the neuroprotective potential of the drug remains unknown. Some of the pharmacological effects of DA stabilizers on DA transmission could be neurotrophic and neuroprotective for neuronal cells as they interact with a number of pathways involved in cell survival and proliferation 12. Although pridopidine binds to striatal DA D2 receptor both *in vivo* 13 and *in vitro* 14, its functional effects are not confined to alterations originating from the DA pathway 6,15.

Recently, it has been reported that pridopidine displays nanomolar affinity at the sigma-1-receptor (Sig-1R) 16, a two-transmembrane domain protein, widely distributed in different regions of the central nervous system (CNS), with involvement in memory, emotion, sensory and motor function tasks 17,18. Sig-1R is a novel molecular chaperone regulating protein folding and degradation at the endoplasmic reticulum (ER) 19. Its selective agonism may ameliorate the accumulation of misfolded proteins in the CNS 20 and increase cell survival in a HD cell model 21. The primary objective of our study was, therefore, to explore the potential neuroprotective effect of pridopidine in pre-clinical HD models, thus providing evidence that might support a potential disease-modifying action of the drug and possibly clarifying other aspects of pridopidine mode-of-action. Using comprehensive outcome measures to assess efficacy, we found that administration of pridopidine induced an overall improvement of motor performance in HD R6/2 mice and exerted anti-apoptotic effect in *in vitro* system. The *in vivo* benefits of the treatment were associated with increased striatal brain derived neurotrophic factor (BDNF), DA, and cAMP-regulated neuronal phosphoprotein (DARPP32) levels, and reduction of mHtt aggregates size. Moreover, our study advances the involvement of Sig-1R in such beneficial effect.

## Materials and methods

### Chemicals

Pridopidine was provided by Neurosearch (NEUR, Copenhagen, Denmark). NE100 was purchased from Santa Cruz Biotechnology, Inc. (Heidelberg Germany) and dissolved according to the manufacturer's instructions.

### Animal models

All *in vivo* experiments were conducted in R6/2 transgenic mice expressing exon 1 of human Htt with approximately 160 ± 10 (CAG) repeats and manifesting first symptoms around week 7, and in wild-type (WT) littermates maintained on the B6CBA strain (Jackson Laboratories, Bar Harbor, MI, USA). Animals were housed singly and maintained under a 12-hr light/dark cycle environment in a clean facility and given free access to food pellets and water. Experimenters were blind to either the genotype of the mice or to the treatment. A total of 60 R6/2 mice and 50 WT littermates were used in this study. Mice from the same F generation were assigned to experimental groups, such that age and weight were balanced. Biochemical and histological experiments were carried out on mice brain tissues, euthanized at fixed time points. R6/2 mice used for testing the effect of pridopidine on animal lifespan died naturally. All experimental protocols were approved by IRCCS Neuromed Animal Care Review Board and by ‘Ministero della Salute’ (permit number: 43/2011-A).

### *In-vivo* drug administration

Pridopidine was dissolved in saline (vehicle), and administered daily by intraperitoneal (i.p.) injection at a dose of 5 or 6 mg/kg per bodyweight during the light phase of the circadian rhythm. Control mice (WT and R6/2) were injected daily with the same volume of vehicle. All the mice were singly housed in home cage. Pridopidine (5 mg/kg) was administered to pre-symptomatic mice starting at week 5 to week 11 (6 week duration) and for symptomatic animals starting from week 7 to week 9 (3 weeks duration) and 1 week of daily administration (6 mg/kg) at week 10.

### Motor behaviour tests and survival study

Training and baseline testing for motor function were carried out prior topridopidine administration. For this purpose, 8–10 mice per group were analysed. General motor function was measured once a week during the entire period of the treatment. Locomotor behaviour and motor performance were performed using the open field and the horizontal ladder task, respectively, as previously described 22.

Spontaneous locomotion and general motor activity were analysed in the open field. Mice was placed in the centre of a square arena and allowed to explore it for 5 min. Quantitative analyses were performed on the total distance travelled. The arena was cleaned with 10% ethanol between each animal. Skilled walking, limb placement and limb coordination were all assessed by the ladder rung walking task. Mice were placed upon a horizontal ladder and the number of times the animal missteps through the ladder (total error score) was evaluated by using a well-established footfall scoring system 23. All tests took place during the light phase of the light–dark cycle and all tests were carried out blindly to the treatment. All mouse cages were daily examined in order to determine lifespan.

### Brain pathology and immunohistochemistry

Wild-type and R6/2 mice were sacrificed by cervical dislocation; brains were surgically removed from the skull and trimmed by removing the olfactory bulbs and spinal cord. The remaining brain were then weighed (in mg), processed and embedded in paraffin wax, and 10 μm coronal sections were cut on an RM 2245 microtome (Leica Microsystems, Srl, Milan, Italy). Five mice/group (*n* = 5) were used and four coronal sections spread over the anterior-posterior extent of the brain (100–200 μm inter-section distance) were scanned. For each coronal section, a total number of 10 fields at 63× magnification was randomly acquired and then analysed. Immunostaining for mutant Htt aggregates was carried out using EM48 antibody (1:100; Millipore, Darmstadt, Germany) 24. Htt inclusions were defined as EM48-positive staining at the light microscope level. The average area of striatal mHtt aggregates per brain section, for each mouse brain, was quantitated by ImageJ software and reported as mHtt aggregates area (μm^2^) 25.

#### Protein lysate preparation

Analysis of variation of protein expression after pridopidine administration was performed by biochemical assays on brain regions. Dissected brain tissues were snap frozen in liquid N2 and pulverized in a mortar with a pestle. Pulverized tissue was homogenized in lysis buffer containing 20 mM Tris, pH 7.4, 1% Nonidet P-40, 1 mM EDTA, 20 mM NaF, 2 mM Na3V04 and 1:1000 protease inhibitor mixture (Sigma-Aldrich Corporation, Buchs SG, Switzerland), sonicated with 2 × 10 sec. pulses and then centrifuged for 10 min. at 10,000 × g.

### Cell models

Conditionally immortalized mouse striatal knock-in cells expressing endogenous levels of wild-type (STHdh^7/7^) or mHtt (STHdh^111/111^) were purchased from the Coriell Cell Repositories (Coriell Institute for Medical Research, Camden, NJ, USA) and were maintained as previously described 26.

#### Analysis of apoptosis

Different concentrations of pridopidine (100, 150, 200 and 300 μM) were tested to investigate the anti-apoptotic effect of the molecule on immortalized cells cultured in serum-free medium at 39°C for six hours. In NE100 experiments, cells were pre-incubated with the compound (10 μM) for 2 hrs before culturing them in apoptotic conditions. At the end of each treatment, cells were collected and incubated with FITC-conjugated Annexin V (Southern Biotech, Birmingham, AL 35209, USA) according to the manufacturer's instructions. Fluorescence Activated Cell Sorting (FACS) analysis was performed as previously described 26.

#### Lysates preparation

Cells were cultured for 5 hrs at 33°C in serum-free medium then treated with 150 μM pridopidine for 10 min. and lysate in lysis buffer containing 20 mM Tris, pH 7.4, 1% Nonidet P-40, 1 mM EDTA, 20 mM NaF, 2 mM Na3V04 and 1:1000 protease inhibitor mixture (Sigma-Aldrich), sonicated with 2 × 10 sec. pulses and then centrifuged for 10 min. at 10,000 × g. In NE100 experiments, cells were pre-incubated with the compound for 2 hrs before adding pridopidine.

### Immunoblottings

40 μg of total protein lysate were resolved on SDS-PAGE and immunoblotted with specific antibodies. Anti-phospho-ERK (1:1000) and anti-ERK (1:1000) (all from cell signaling) were used for analysis of the kinase activation. For the analysis of DARPP-32, BDNF expression total lysate were immunoblotted with the anti-DARPP-32 (1:1000; Cell Signaling), anti-BDNF (1:500; Santa Cruz Biotechnology), respectively. Mutant huntingtin aggregates were detected using EM48 antibody (1:1000; Millipore). Anti-aTubulin (1:5000; Abcam, plc, Cambridge, UK) or anti-β-actin (1:3000; Sigma-Aldrich) antibodies were used for protein normalization. HRP-conjugated secondary antibodies (GE Healthcare, Buckinghamshire, UK) were used at 1:5000 dilution. Protein bands were detected by ECL Prime (GE Healthcare) and quantitated with Quantity One (Bio-Rad Laboratories) and/or ImageJ software.

### Statistics

Two-way anovas followed by Bonferroni post-test for multiple comparisons were used to compare treatment groups in the open field and horizontal ladder tests. Kaplan–Meier curves employing Log-Rank Test were used to analyse mice survival. Non-parametric Mann–Whitney *U* was used to analyse cell survival, mouse brain weight, BDNF, DARPP-32 protein levels. Two-tailed *t*-tests were employed in all other experiments. All data are expressed as mean ± SD.

## Results

### Pridopidine improves motor function and prolongs life-span in pre-symptomatic HD mice

**Figure 1:**
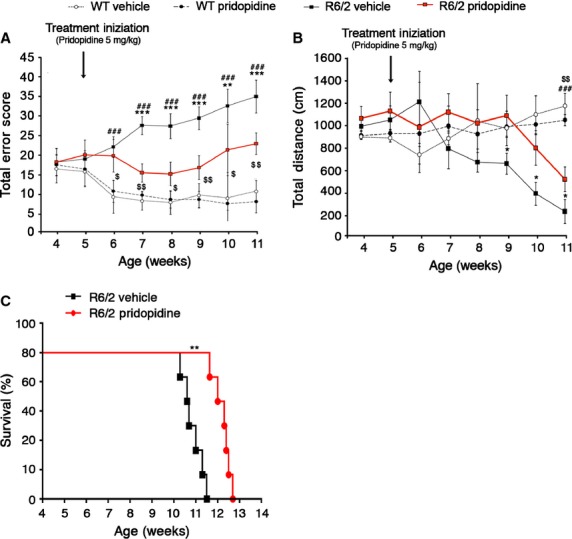
Chronic administration of pridopidine (5 mg/kg) improves motor function and prolongs lifespan in pre-symptomatic R6/2 mice. (A) Analysis of motor coordination, reported as total error score, on horizontal ladder task before and after the treatment in 5 week-old R6/2 mice and WT littermates. Total Error Score is the cumulative number of footfall errors in the horizontal ladder task. (B) General locomotor activity in the open field in the same mice, before and after treatment. Each data point represents the average performance ± SD of 8–10 mice for each group. **, *P* < 0.001; ***, *P* < 0.0001 (vehicle-treated R6/2 *versus* pridopidine-treated R6/2); ^###^, *P* < 0.001 (vehicle-treated WT *versus* vehicle-treated R6/2); ^$^, *P* < 0.05; ^$$^, *P* < 0.01 (pridopidine-treated WT *versus* pridopidine-treated R6/2, two-way anova with Bonferroni post-test). (C) Kaplan–Meier probability of survival analysis in pridopidine- and vehicle-treated R6/2 mice. *N* = 8–10 mice for each group **, *P* < 0.01 (Log-Rank Test).

To further confirm the beneficial effect of pridopidine on HD motor phenotype and to elucidate whether the molecule may act also as neuroprotective agent, preclinical studies in R6/2 mice have been undertaken. Daily administration of pridopidine at a dose of 5 mg/kg, the most effective dose with no adverse effects (data not shown), starting at the pre-symptomatic stage at 5 weeks for 6 weeks, significantly preserved motor function and prevented the progressive and dramatic motor worsening commonly observed in R6/2 mice (Fig.1A and B). Our results indicate that the beneficial effects of the drug were maintained for about 4 weeks, after which mice showed a slight worsening in performing both the horizontal ladder task and the open field (Fig.1A and B). In addition, according to a Kaplan–Meier survival curve analysis, pridopidine efficiently extended lifespan in the same mice (Fig.1C), suggesting a neuroprotective potential of the drug.

### Pridopidine transiently improves motor function in symptomatic HD mice

**Figure 2:**
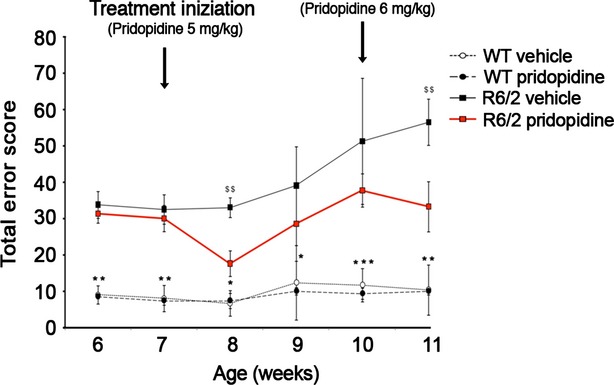
Chronic administration of pridopidine (5 mg/kg) transiently improves motor function in symptomatic R6/2 mice. Analysis of motor coordination, reported as total error score on the horizontal ladder task, before and after the dosage change (started at 10 weeks of age) in R6/2 mice and WT littermates. Each data point represents the average performance ± SD of 8 mice for each group. ^$$^, *P* < 0.001 (vehicle-treated R6/2 *versus* pridopidine-treated R6/2); *, *P* < 0.05; **, *P* < 0.001; ***, *P* < 0.0001 (vehicle- and pridopidine-treated WT *versus* vehicle-treated R6/2, two-way anova with Bonferroni post test).

Next, to investigate whether the administration of pridopidine could exert a similar beneficial effect or restore normal motor function in the advanced stage of HD, symptomatic R6/2 mice with evident compromised motor function were chronically treated for 3 weeks with pridopidine or placebo starting at 7 weeks of age. After a marked improvement in motor symptoms within the first week of treatment, mice appear to become less responsive to the drug, and disease severity increased to be almost indistinguishable from symptomatic vehicle-treated mice (Fig.2).

In this regard, we tested the possibility that an increased dose of pridopidine, 6 mg/kg, which causes hypokinesia if administered at the beginning of the treatment, could re-establish the effectiveness of the drug on motor performance. One week of the 6 mg/kg dose of pridopidine starting week 10 relieved the progression of motor decline and triggered the recovery of motor function in symptomatic R6/2 mice (Fig.2).

### Pridopidine positively modulates the expression of neuroprotective molecules in R6/2 mice

**Figure 3:**
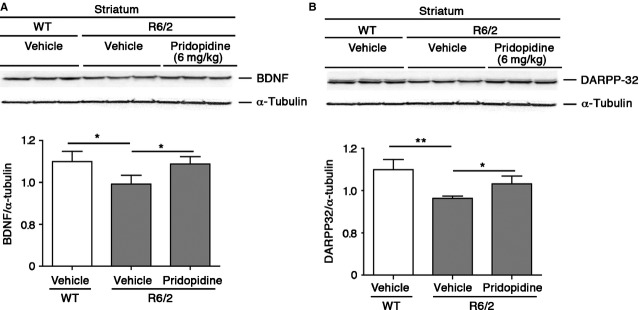
Pridopidine enhances the expression of both BDNF and DARPP32 in the striatum of R6/2 mice. (A) Representative Western Blotting and densitometric analysis of BDNF and (B) DARPP32 protein in striatal tissues from WT littermates and R6/2 mice, before and after the treatment. Data are represented as mean ± SD, *n* = 5 for each group of mice. *, *P* < 0.05; **, *P* < 0.001 (Non-parametric Mann–Whitney *U*).

To explore whether molecular targets (*i.e*. BDNF and DARPP32), normally involved in the control of brain function and homeostasis and, commonly associated with neuroprotection, varied with pridopidine treatment, biochemical analysis were carried out in both pre-symptomatic and symptomatic R6/2 mice. After chronic administration of 5 mg/kg of pridopidine, we did not detect any difference in the expression of BDNF between pridopidine-treated and vehicle-treated mice in striatal or in cortical tissues (Fig. S1A and B). On the other hand, immunoblotting analysis of DARPP32 highlighted a slight increase of protein expression in pridopidine-treated R6/2 mice (Fig. S1C). Although not statistically significant, the tendency towards increased DARPP32 expression is suggestive of a potential neuroprotective action of the drug. To further investigate the neuroprotective properties of pridopidine, we increased the dose of pridopidine (from 5 to 6 mg/kg) and observed, after 1 week of treatment, a restoration of normal expression of both BDNF and DARPP32 protein in the striatal lysate from treated R6/2 mice (Fig.3A and B). No changes of BNDF levels were detected in lysate from cortical tissues (Fig. S2).

### Pridopidine significantly reduces the size of mHtt aggregates in the striatum of R6/2 mice

**Figure 4:**
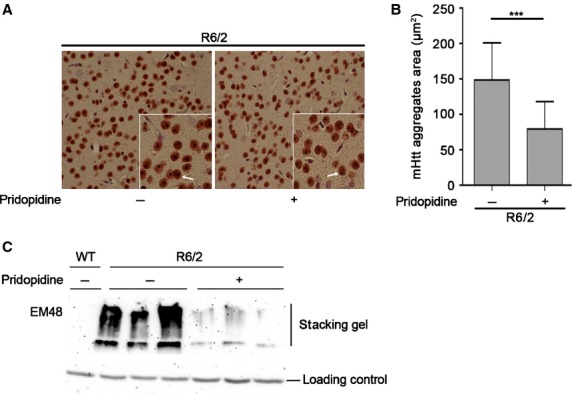
Pridopidine induces remodelling of mHtt aggregates. (A) Representative micrograph of EM48 positive striatal mHtt aggregates from vehicle- and pridopidine-treated R6/2 mice. Arrows indicate mHtt aggregates (scale bar 25 μm). (B) Semiquantitative analysis of mHtt aggregates size (area) for brain section. For each mouse brain, four coronal sections were analysed. Data are represented as mean ± SD. *n* = 5 for each group of mice. ***, *P* < 0.0001 (two-tailed *t*-test). (C) Immunoblotting for EM48 positive mHtt aggregates in the stacking gel in protein extracts from Wt and R6/2 striatal brain tissues before and after the administration of pridopidine.

To elucidate whether pridopidine could have any effect on mHtt toxicity and aggregates formation in the brain, at the end of the treatment R6/2 mice were killed, and immunodetection of mutant protein aggregation in the striatal tissues was performed. Immunostaining analysis with EM48 antibody showed mHtt aggregates in the striatum of presymptomatic R6/2 mice (Fig. S3). Our results revealed a significant reduction in the size of single aggregates in pridopidine-treated R6/2 compared with vehicle-infused mice (Fig.4A and B). The ability of pridopidine to reduce the size of mHtt aggregatewas also confirmed by immunoblotting analysis (Fig.4C). Next, in order to elucidate the widespread effects that pridopidine could have on the brain anatomy, we investigated changes in the brain weight as an index of global disruption of neuronal systems, a common feature of HD. As expected, brains from vehicle-treated R6/2 mice weighed approximately15% less than brains from WT littermates (Fig. S4). Mice chronically treated with pridopidine displayed a slight trend towards the preservation of brain weight loss, however, it did not reach any statistically significant difference compared with vehicle-treated mice (Fig. S4).

### The neuroprotective and the anti-apoptotic effect of pridopidine is mediated by Sig-1R in HD cell model

**Figure 5:**
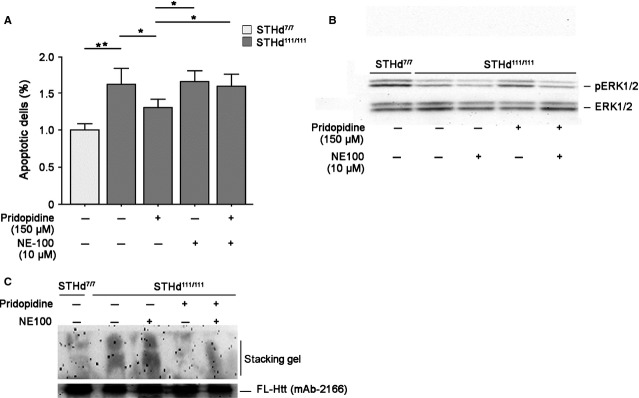
Administration of pridopidine protects HD striatal-derived cell lines from apoptosis and promotes ERK activation. (A) Apoptosis in STHdh cell line cultured for 6 hrs in serum-free medium in presence or absence of 150 μM pridopidine and/or NE100. Data are represented as mean ± SD of three experiments, each performed in triplicate. *, *P* < 0.05; **, *P* < 0.001 (Non-parametric Mann–Whitey *U*). (B) Representative Western Blotting of ERK phosphorylation in protein extracts from cells treated or not with pridopidine and/or NE100. (C) Immunoblotting for EM48 positive mHtt aggregates in the stacking gel in cell lysates cultured in presence or absence of pridopidine and/or NE100.

To clarify the neuroprotective efficacy of pridopidine and to explore the potential underling molecular mechanism, we evaluated the ability of the drug to protect cells from apoptosis and to eventually activate pro-survival targets. Administration of pridopidine (150 μM), the most effective dose (Fig. S5), significantly reduced apoptosis in immortalized striatal knock-in cells expressing endogenous levels of mutant Htt (STHdh^111/111^) (Fig.5A) and markedly enhanced phosphorylation state of prosurvival kinase ERK (Fig.5B). The neuroprotective effect of pridopidine and its ability to promote activation of pro-survival pathways *in vitro* was completely abolished in presence of NE100, a selective antagonist of Sig-1R, suggesting that thelatter plays a key role in determining the effectiveness of pridopidine in HD cells (Fig.5A and B). Moreover, the specific antagonism of Sig-1R partially prevented pridopidine-induced reduction in the size of mHtt aggregates in HD cells (Fig.5C).

## Discussion

Dopamine imbalance plays a key role in the pathophysiology of a number of neuropsychiatric and neurodegenerative diseases 27, including HD 2. Dopamine alterations have been reported in mouse models of HD 28,29 and post-mortem tissues from HD patients 30,31 and may account for both motor and non-motor manifestations of the disease. There is evidence of a progressive reduction of striatal DA levels concomitant with motor abnormalities in both systems.

Dopaminergic input is crucial for the regulation of corticostriatal synaptic transmission and since the abnormalities in the DA system appear to underlie many of the behavioural symptoms of HD, treatment with modulators of dopaminergic neurotransmission may have therapeutic value for the disease. Current treatment options for HD are confined to anti-dopaminergic agents, often accompanied with serious side effects 32,33. However, promising research on the development of DA-stabilizer molecules, offers new hope. Pridopidine, a DA stabilizer, is currently under evaluation in the Pride-HD trial, for symptomatic treatment of HD, after the compound completed a previous Phase III clinical study in Europe (MermaiHD study) and a Phase II study in the United States (HART study) 9,11. Both studies showed a tendency towards improvement very close to significance in the primary endpoint of modified motor score (mMS) and found positive effects on the secondary endpoint of the TMS of the UHDRS 34. Patients receiving pridopidine displayed these improvements in their motor symptoms without deleterious side effects 10. The discovery of such beneficial effect of pridopidine on motor function supports the molecule as potential novel therapy and corroborates the hypothesis that pridopidine may have disease-modifying properties in HD.

What makes pridopidine unique is that, depending on the levels of DA in the cells, it will either have stimulatory or inhibitory effects 35. It is, however, challenging to determine whether the clinical benefits of pridopidine are due to short-term symptomatic effects or to the potential long-term neuroprotective properties of the drug.

To this purpose, we have undertaken pre-clinical studies both in *in vivo* and *in vitro* models of HD. Treatment with pridopidine significantly improved the overall neurological phenotype in R6/2 mouse model and the therapeutic benefits seen across outcome measures, included improvement of motor performance, increased survival and neuroprotection.

Intriguingly, in this study we observed an interesting link between pridopidine dose and beneficial effects in R6/2 mice. The initial dose of pridopidine, 5 mg/kg, after the first few weeks of therapeutic efficacy, became less effective and an increased dose, 6 mg/kg was required to maintain the beneficial effect. The optimal dose of pridopidine had been established in a pilot study, during which different doses of the drug (2.5, 5.0, 6.0 and 10 mg/kg) were daily administered. When given at high doses (10 mg/kg), pridopidine induced side effects such as rigidity and akinesia (data not shown). A dose of 5 mg/kg pridopidine was found to be the most effective one with no adverse effects. The slightly higher dose, 6 mg/kg pridopidine was found to be deleterious to some aspects of motor performance when administered as starting dose (Fig. S6); although not exactly understandable, these observations prompt us to argue that the choice of dosage is critical and further studies are necessary to determine whether the varying effects of the pridopidine doses may reflect the dynamic and time-dependent changes that occur in the DA system as the HD disease progresses.

Furthermore, beside confirming the potential of pridopidine to represent a symptomatic treatment in HD, our findings highlight a neuroprotective action of the drug; administration of pridopidine increased the expression of both BDNF and DARPP32, proteins normally implicated in neuronal health 36,37 and reduced in animal models of HD and post-mortem samples of HD patients 38, and ameliorated mHtt aggregation, commonly linked to mHtt toxicity 39 Consistently, pridopidine protected HD cells from apoptosis and promoted the activation of pro-survival kinase ERK. Similar to (-)-OSU6162, another dopaminergic stabilizer recently reported in a HD study 12, pridopidine exerted its antiapoptotic effect only at concentrations of 150 μM. Concentrations other than 150 μM may possibly activate additional pathways that could counteract the pro-survival action of the drug.

From mechanistic standpoint, our data imply that the Sig-1R is involved in the mechanism of action of pridopidine and suggests that the anti-apoptotic effect of pridopidine *in vitro* may depend on the stimulation of Sig-1R. In support of this, we have found that the pharmacological blockage of Sig-1R, by a selective antagonist NE100, completely abolished cell survival and ERK activation mediated by pridopidine, *in vitro*. Based on these observations, to understand the neuroprotective properties of pridopidine, other targets beside DA receptors should be considered. The involvement of Sig-1R may help clarify the putative mechanisms of action through which pridopidine exerts its therapeutic action; however, further studies are warranted in support of our hypothesis.

In summary, based on our finding, we hypothesize that pridopidine may be a neuromodulatory agent with neuroprotective properties in HD. It may however, lose its effectiveness over the time and dose variations may be theoretically required throughout treatment to maximize the efficacy of the drug, at least in R6/2 mouse model. Importantly, we believe that further study is needed to adequately address this specific issue and to clearly define the potential neuroprotective properties of pridopidine in the R6/2 mice model.

## Supporting Information

**Figure S1** Chronic administration of pridopidine at constant dose fails to enhance cerebral expression of both BDNF and DARPP32. **Figure S2** Change of pridopidine dose does not increase expression of BDNF in the cortex of R6/2 mice. **Figure S3** mHtt aggregates are detectable at presymptomatic stage of the disease. **Figure S4** Chronic administration of pridopidine fails to prevent loss of brain weight in R6/2 mice. **Figure S5** Pridopidine (150 μM) resulted to be the effective dose in protecting HD cells from apoptosis. **Figure S6** Chronic administration of pridopidine (6 mg/kg) induces hypokinesia in R6/2 mice.
