# Supplementary material for: Pridopidine, a dopamine stabilizer, improves motor performance and shows neuroprotective effects in Huntington disease R6/2 mouse model
Source: J Cell Mol Med. 2015 Jun 22;19(11):2540–8. doi: 10.1111/jcmm.12604 (PMC4627560; doi:10.1111/jcmm.12604)
Supplement: Supplementary file 1 — Figure S1 Chronic administration of pridopidine at constant dose fails to enhance cerebral expression of both BDNF and DARPP32. Figure S2 Change of pridopidine dose does not increase expression of BDNF in the cortex of R6/2 mice. Figure S3 mHtt aggregates are detectable at presymptomatic stage of the disease. Figure S4 Chronic administration of pridopidine fails to prevent loss of brain weight in R6/2 mice. Figure S5 Pridopidine (150 μM) resulted to be the effective dose in protecting HD cells from apoptosis. Figure S6 Chronic administration of pridopidine (6 mg/kg) induces hypokinesia in R6/2 mice. [file jcmm0019-2540-sd1.pdf]

Supplementary Figure 1

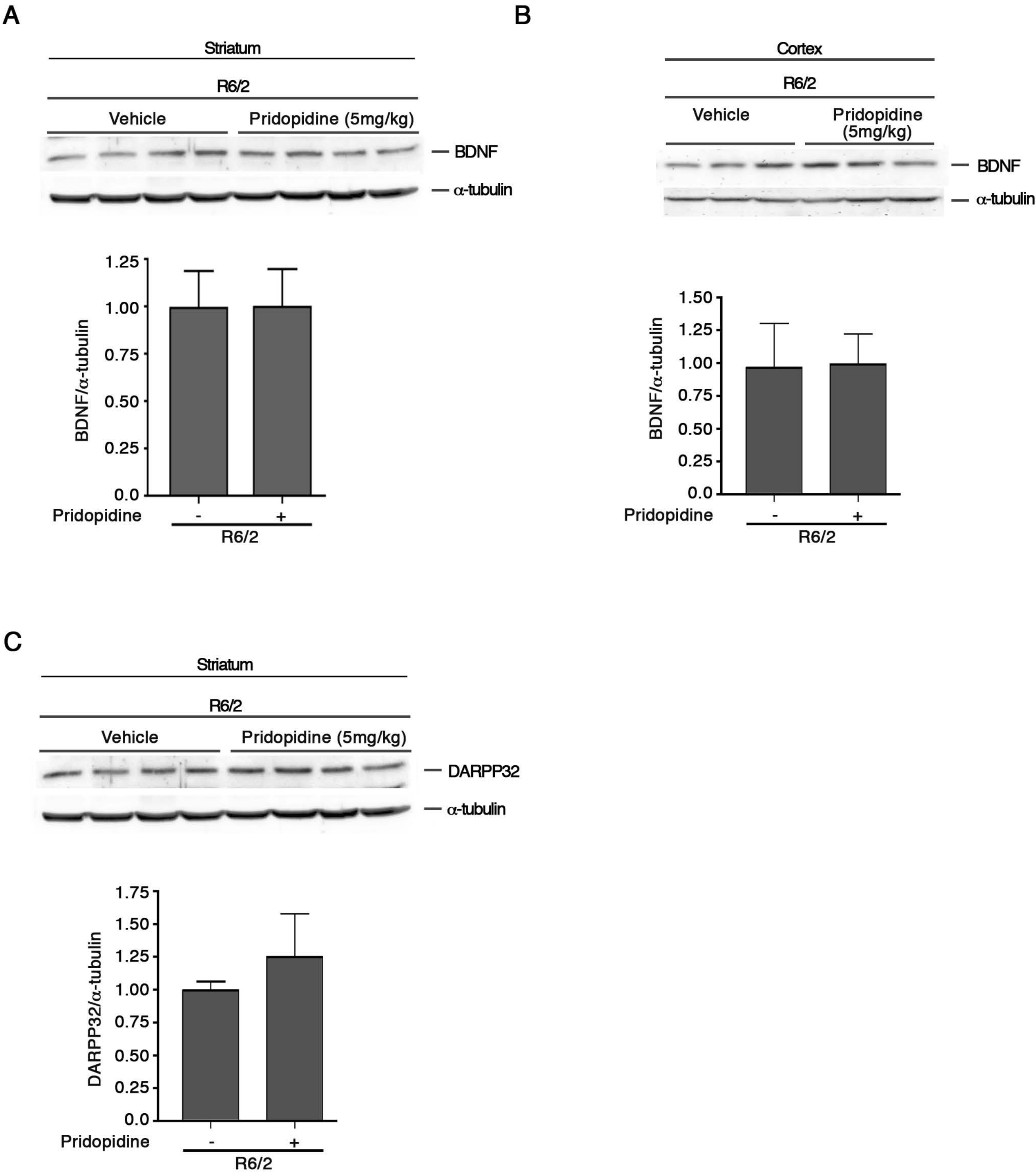

Supplementary Figure 2

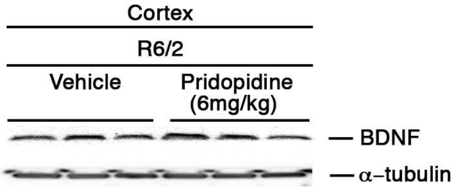

Supplementary Figure 3

R6/2 Striatum

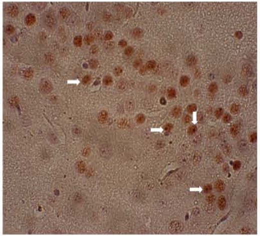

EM48 positive cells

Supplementary Figure 4

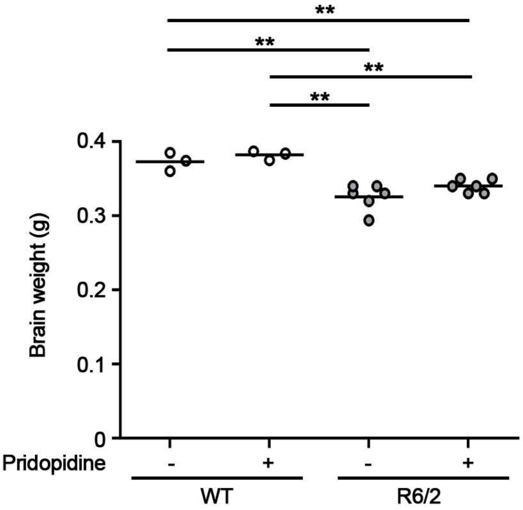

Supplementary Figure 5

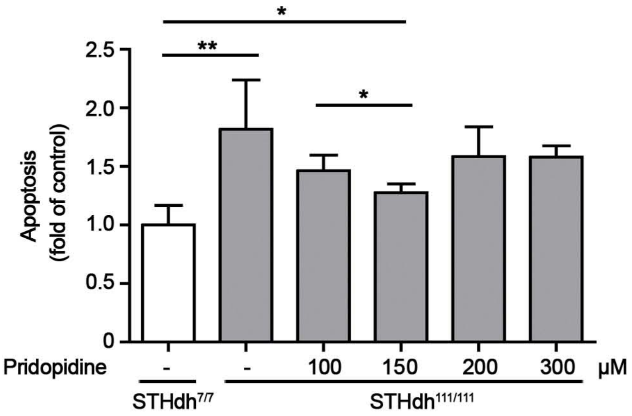

Supplementary Figure 6

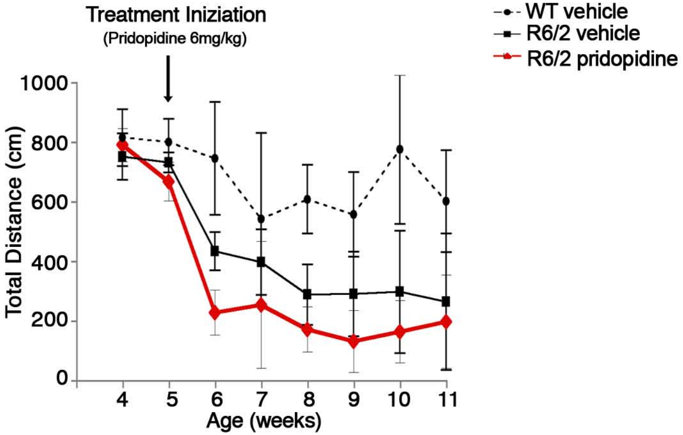

Supplementary Figure 1. Chronic administration of pridopidine at constant dose fails to enhance cerebral expression of both BDNF and DARPP32. (**A-B**) Representative Western Blottings and densitometric analyses of BDNF protein expression in striatal and cortical tissue of vehicle- and pridopidine-treated R6/2 mice. (**C**) Representative Western Blotting and densitometric analysis of DARPP32 in striatal tissues of the same mice. Data are represented as mean ± SD. n=5 for each group of mice.

Supplementary Figure 2. Change of pridopidine dose does not increase expression of BDNF in the cortex of R6/2 mice. Representative Western Blotting of BDNF protein expression in cortical tissue of vehicle- and pridopidine-treated R6/2 mice. Data are represented as mean ± SD, n=5 for each group of mice.

Supplementary Figure 3. mHtt aggregates are detectable at presymptomatic stage of the disease. Representative micrograph of EM48 positive cells from WT and R6/2 mouse striatum. Arrows indicate mHtt aggregates.

Supplementary Figure 4. Chronic administration of pridopidine fails to prevent loss of brain weight in R6/2 mice. Average brain weight of vehicle- and pridopidine -treated WT and R6/2 mice. Vehicle- and pridopidine-treated WT mice n=5 for each group; vehicle-treated R6/2 mice n=7; pridopidine-treated mice n=7, **, $p<0.001$ (Non-parametric Mann Whitey U)

Supplementary Figure 5. Pridopidine (150µM) resulted to be the effective dose in protecting HD cells from apoptosis. Apoptosis in striatal-derived cell lines cultured for six hours in serum-free medium in presence or absence of different concentrations of pridopidine. Data are represented as the mean ± SD of

two independent experiments performed in quadruplicate**.** *, $p<0.05$; **, $p<0.001$ (non parametric Mann Whitney U)

Supplementary Figure 6. Chronic administration of pridopidine (6mg/kg) induces hypokinesia in R6/2 mice. General locomotor activity in the open field in the R6/2, before and after treatment and WT littermates. Each data point represents the average performance ± SD of 3 mice for each group.
